# Leucocyte telomere length and conduction system ageing

**DOI:** 10.1136/heartjnl-2024-324875

**Published:** 2024-12-17

**Authors:** Stefan van Duijvenboden, Christopher P Nelson, Zahra Raisi-Estabragh, Julia Ramirez, Michele Orini, Qingning Wang, Nay Aung, Veryan Codd, Svetlana Stoma, Elias Allara, Angela M Wood, Emanuele Di Angelantonio, John Danesh, Nicholas C Harvey, Steffen E Petersen, Patricia B Munroe, Nilesh J Samani

**Affiliations:** 1 Nuffield Department of Population Health, University of Oxford, Oxford, UK; 2 William Harvey Research Institute, Barts and The London Faculty of Medicine and Dentistry, Queen Mary University of London, London, UK; 3 Institute of Cardiovascular Science, UCL, London, UK; 4 Department of Cardiovascular Sciences, University of Leicester, Leicester, UK; 5 NIHR Leicester Biomedical Research Centre, Glenfield Hospital, Leicester, UK; 6 NIHR Barts Biomedical Research Centre, Queen Mary University of London, London, UK; 7 Barts Heart Centre, St Bartholomew’s Hospital, Barts Health NHS Trust, London, UK; 8 Aragon Institute of Engineering Research, University of Zaragoza, Zaragoza, Spain; 9 Biomedical Research Networking Center for Bioengineering Biomaterials and Nanomedicine, University of Zaragoza, Zaragoza, Spain; 10 Department of Public Health and Primary Care, University of Cambridge, Cambridge, UK; 11 Victor Phillip Dahdaleh Heart and Lung Research Institute, University of Cambridge, Cambridge, UK; 12 NIHR Blood and Transplant Research Unit in Donor Health and Behaviour, University of Cambridge, Cambridge, UK; 13 Health Data Science Research Centre, Human Technopole, Milan, Italy; 14 Department of Human Genetics, Wellcome Sanger Institute, Hinxton, UK; 15 MRC Lifecourse Epidemiology Unit, University of Southampton, Southampton, Hampshire, UK; 16 NIHR Southampton Biomedical Research Centre, University of Southampton and University Hospital Southampton NHS Foundation Trust, Southampton, UK

**Keywords:** Electrophysiology, Genetics, Epidemiology

## Abstract

**Background:**

Deterioration of the cardiac conduction system is an important manifestation of cardiac ageing. Cellular ageing is accompanied by telomere shortening and telomere length (TL) is often regarded as a marker of biological ageing, potentially adding information regarding conduction disease over and above chronological age. We therefore sought to evaluate the association between leucocyte telomere length (LTL) on two related, but distinct aspects of the cardiac conduction system: ECG measures of conduction (PR interval and QRS duration) and incident pacemaker implantation in a large population-based cohort.

**Methods:**

In the UK Biobank, we measured PR interval and QRS duration from signal-averaged ECG waveforms in 59 868 and 62 266 participants, respectively. Incident pacemaker implantation was ascertained using hospital episode data from 420 071 participants. Associations with LTL were evaluated in (Cox) multivariable regression analyses adjusted for potential confounders. Putative causal effects of LTL were investigated by mendelian randomisation (MR).

**Results:**

Mean PR interval and QRS duration were 144.2 ms (± 20.4) and 92.3 ms (± 7.8), respectively, and there were 7169 (1.7%) incident pacemaker implantations, during a median follow-up period of 13.6 (IQR 1.5) years. LTL was significantly associated with PR interval (0.19 ms (95% CI: 0.03 to 0.35), per 1 SD shorter LTL, p=0.021), but not QRS duration. After adjusting for age, sex and cardiovascular risk factors, shorter LTL remained associated with an increased risk for incident pacemaker implantation (HR per SD decrease in LTL: 1.03 (95% CI: 1.01 to 1.06), p=0.012). MR analysis showed a trend towards an association of shorter LTL with longer PR interval and higher risk of pacemaker implantation but was likely to be underpowered.

**Conclusions:**

Shorter LTL was significantly, and possibly causally, associated with prolongation of atrioventricular conduction and pacemaker implantation, independent of traditional cardiovascular risk factors. Our findings support further research to explore the role of ageing on cardiac conduction beyond chronological age.

**WHAT IS ALREADY KNOWN ON THIS TOPIC**

- Deterioration of the cardiac conduction system is an important manifestation of cardiac ageing. While ageing is commonly defined by chronological age, a great heterogeneity in cardiac ageing trajectories occurs in individuals of the same age.

**WHAT THIS STUDY ADDS**

- This is the largest population-based study to date to examine the impact of cellular ageing, measured by leucocyte telomere length (TL), on the deterioration of cardiac conduction. We found evidence that shorter TL is associated with a longer ECG PR interval and future de novo pacemaker insertion, independent of other cardiovascular risk factors.

**HOW THIS STUDY MIGHT AFFECT RESEARCH, PRACTICE OR POLICY**

- Our findings indicate a potential role for cellular ageing in the pathogenesis and clinical presentation of atrioventricular conduction disease.

## Introduction

 Deterioration of the cardiac conduction system is an important manifestation of cardiac ageing, which includes increased incidence of sinus node dysfunction, conduction delay or block at the atrioventricular (AV) node and/or within the His-Purkinje system.^1^,^4^ Failure of AV conduction may result in syncope and significant associated injuries. Thus, high-grade AV block is an indication for permanent pacemaker implantation.

While ageing is commonly defined by chronological age, a great heterogeneity in ageing trajectories and health outcomes occurs in individuals of the same age.^5^ Measures of biological age may provide added information about the impacts of ageing independent of chronological age.^6^ This knowledge could be helpful in defining potential indicators of conduction system ageing, which is key for improved risk stratification and understanding of disease mechanisms. Telomeres are repetitive DNA sequences located at the ends of chromosomes, which progressively shorten in somatic cells with increasing number of cell divisions and have therefore been regarded as a marker of biologic ageing. Previous work has shown that telomere length (TL) may provide insights into ageing across key organ systems beyond chronological age.^7 8^ Whether variation in TL is also associated with deterioration of the cardiac conduction system remains unclear.

In this study, we therefore examined the association between leucocyte telomere length (LTL), a practical indicator of TL that correlates well across various tissues,^9^ and cardiac conduction in the large population-based cohort of UK Biobank. Specifically, we studied two related, but distinct, aspects of the cardiac conduction system—one reflected by electrical measures of cardiac conduction (electrocardiographic PR interval and QRS duration) and the other reflected by a significant clinical outcome of cardiac conduction deterioration: incident pacemaker implantation. Prolongation of both PR interval and QRS duration are associated with increased risk of future permanent pacemaker insertion in the general population.^4 10^

## Methods

### Participants

**Figure 1:**
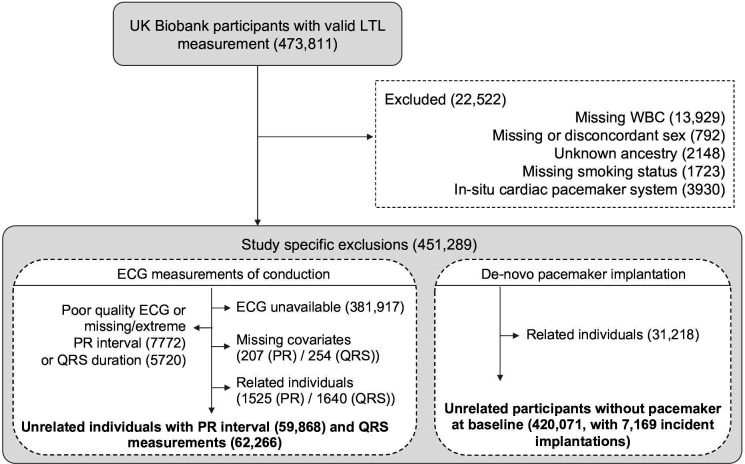
Exclusion diagrams for PR interval, QRS duration and pacemaker implantation cohorts. LTL, leucocyte telomere length; WBC, white blood cell count.

From the 473 811 participants in UK Biobank with valid measurements of LTL,^11^ we excluded all participants with missing leucocyte count, mismatches between reported and genetic sex and unknown ancestry (figure 1). We also excluded participants with evidence of pre-existing pacemakers at baseline as this could render invalid inferences regarding ECG conduction measures and because we were specifically interested in incident pacemaker implantation. From the remaining participants (n=452 997), we created two cohorts: one to study ECG measurements of cardiac conduction and another to study incident pacemaker implantation (figure 1). Please note that both cohorts were not mutually exclusive but were constructed to study different aspects of the cardiac conduction system in parallel.

In the ECG cohort, we included 69 625 participants (figure 1) with single-lead (Lead I) ECG scans (CAM-USB V.6.5, CardioSoft V.6.51) available at baseline. Signal-averaged waveforms recorded during a 15 s resting period were analysed for PR interval and QRS duration. Following exclusions for poor quality and extreme measurements (PR interval<110 ms or >200 ms; QRS duration<80 ms or >120 ms) and genetic relatedness (figure 1), there were 59 868 and 62 266 participants included for analysis of PR interval and QRS duration, respectively. Genetically unrelated samples were obtained by randomly excluding one from each pair based on a kinship coefficient of K>0.088. In the pacemaker implantation cohort, there were 420 071 genetically unrelated individuals with hospital follow-up data included in the analysis (figure 1). Cases of incident pacemaker implantation for a bradycardia indication were ascertained using International Classification of Disease and OPCS Classification of Surgical Operations and Procedures V.4 codes from the hospital episode statistics (HES). Details are provided in online supplemental tables 1 and 2.

### LTL measurement

Details on the process of measuring of LTL, the extensive quality checks and the adjustment for technical factors have been reported previously.^11^ In brief, LTL was measured as the ratio of telomere repeat copy number (T) relative to that of a single copy gene (S, HBG) from the peripheral blood leucocyte DNA, extracted from blood collected at baseline, using a multiplex quantitative PCR method. LTL values were log_e_ transformed and Z standardised for all analyses.

### Statistical analyses

The descriptive statistics are presented as mean±SD for continuous variables and frequency (percentage) for categorical variables. The trends across PR and QRS quintiles were examined using Cuzick’s extension^12^ of the Wilcoxon rank-sum test for continuous variables and the χ^2^ test for trend for ordinal variables. We removed the confounding influence of chronological age at baseline, white blood cell count and self-reported ethnicity by taking the residuals of log_e_ LTL regressed on these variables. The associations between log_e_ LTL residuals and ECG conduction measures were evaluated in multivariable linear regression models adjusted for sex, heart rate, height and body mass index (BMI). The association between log_e_ LTL residuals and incident pacemaker implantation was evaluated using Cox proportional hazards regression adjusted for sex. Time-to-event duration was obtained from the admission date recorded in the HES. The censor date for HES data was 31 October 2022, the median (IQR) follow-up time was 13.6 (IQR: 1.5) years. Proportional hazards assumptions were assessed and met (online supplemental figure 1). Significant associations for ECG measures and pacemaker implantation were additionally adjusted for traditional cardiovascular risk factors, including current smoking, hypertension, type 2 diabetes mellitus and prevalent coronary artery disease (definitions provided in online supplemental table 3), to assess their potential confounding effects. The effect sizes and hazard ratios were represented per SD LTL shortening. A p value less than 0.05 was considered statistically significant. All analyses were conducted in R V.4.2.0.^13^

### Sensitivity analysis

As cardiac conduction can be affected by vagal tone or antiarrhythmic medication, post hoc sensitivity analyses were performed to explore whether associations were affected by these parameters. The influence of vagal tone was explored in a subgroup of individuals with ECG recordings who also participated in an exercise test immediately after conduction measurements were taken. Vagal tone was measured by the speed of heart rate recovery after exercise^14^ and included as an additional covariate in the multivariable linear regression models. The influence of antiarrhythmic medication was explored by excluding participants exposed to beta and calcium blockers.

### Mendelian randomisation (MR) analysis

To examine potential causality of LTL with observationally associated cardiac conduction traits (PR interval and pacemaker implantation), we conducted single-sample univariable MR using two-sample methods shown to be robust in large-scale biobanks.^15^ The inverse-variance weighted method^16^ was implemented to test for the possible causal effect of LTL on PR interval and pacemaker implantation, based on 130 independent and pleiotropically pruned variants associated with LTL.^8^ The PR interval estimates were obtained from previously performed meta-analysis by our group, including 293 051 individuals of European ancestry.^17^ For pacemaker implantation, effect estimates were obtained by performing logistic regression between pacemaker implantation and the allele dosage information available in UK Biobank. Sensitivity analyses for MR were performed using (1) MR-Egger regression to estimate unmeasured pleiotropy in the intercept,^18^ (2) weighted median estimator to assess the robustness to extreme single-nucleotide polymorphism (SNP)–outcome associations^19^ and (3) a contamination mixture method to explore potential presence of multiple pathways.^20^

## Results

### Baseline population characteristics

**Figure 2:**
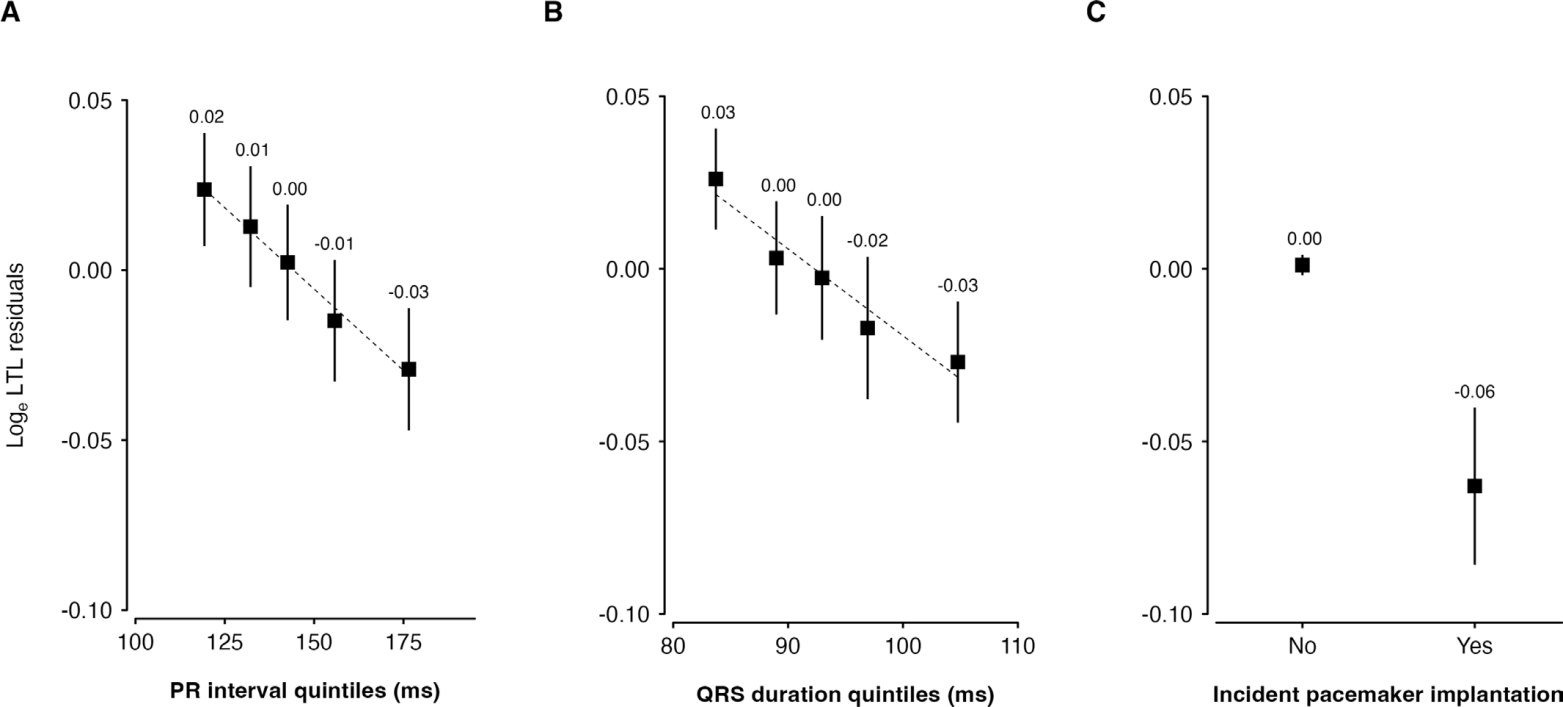
Leucocyte telomere length (LTL) plotted as function of PR interval quintiles (A), QRS duration (B) and de novo pacemaker implantation (C). We removed the confounding influence of chronological age, white blood cell count and self-reported ethnicity by taking the residuals of log_e_ LTL regressed on these variables. Trends across PR interval quintiles, QRS duration quintiles and pacemaker implantation outcome were both significant (p<0.001).

In the ECG cohort (n=59 868 and 62 266 for PR interval and QRS duration, respectively), the average age±SD was: 56.8±8.1 years for participants with PR interval and QRS duration available and approximately 46% were men (tables1  2). The average PR interval and QRS duration were 144.2±20.4 and 92.3±7.8 ms, respectively. Individuals in the higher PR interval quintiles were more likely to be chronologically older and men with higher prevalence of cardiovascular risk factors (table 1). A similar trend was observed for QRS duration. LTL decreased with increasing PR interval and QRS duration quintiles (figure 2A,B). In the pacemaker cohort, there were 7169 (1.7%) incident pacemaker implantations. Individuals who received a pacemaker implantation, as compared with those who did not, were older, more likely to be men (table 3), and had shorter LTL (figure 2C).

**Table 1:** Baseline characteristics according to quintiles of PR interval

Variable	Full cohort (59 868)	PR interval quintile levels					
		1st (<126 ms)	2nd (126–136 ms)	3rd (136–148 ms)	4th (148–162 ms)	5th (>162 ms)	P value
Chronological age (years)	56.8 (8.1)	56.0 (8.3)	56.3 (8.2)	56.7 (8.0)	57.1 (7.9)	58.0 (7.8)	<0.001
Men (%)	46.4	36.9	42.5	46.2	51.3	57.3	<0.001
WBC (mmol/L)	7.1 (2.1)	7.2 (2.1)	7.1 (2.1)	7.1 (1.9)	7.0 (1.8)	7.0 (2.4)	<0.001
Height (cm)	168.7 (9.3)	167.4 (9.1)	168.1 (9.1)	168.5 (9.2)	169.5 (9.4)	170.3 (9.2)	<0.001
BMI	27.5 (4.7)	26.6 (4.4)	27.3 (4.6)	27.6 (4.6)	27.9 (4.7)	28.3 (4.8)	<0.001
LTL	0.0 (1.0)	0.0 (1.0)	0.0 (1.0)	0.0 (1.0)	0.0 (1.0)	−0.1 (1.0)	<0.001
Heart rate (bpm)	71.2 (11.8)	73.7 (11.7)	72.7 (11.6)	71.4 (11.4)	69.7 (11.5)	68.0 (11.9)	<0.001
Coronary artery disease (%)	3.3	2.1	2.5	2.9	3.8	5.6	<0.001
Hypertension (%)	53.6	49.9	51.6	53.6	54.6	59.0	<0.001
Active smoker (%)	9.3	10.2	10.3	9.2	8.4	8.3	<0.001
Diabetes mellitus (%)	5.5	4.6	4.7	5.8	6.1	6.4	<0.001
Ancestry (%)							
Asian	3.2	3.9	3.4	3.5	2.6	2.3	<0.001
Black	3.0	2.7	2.5	2.7	3.3	3.7	<0.001
Chinese	0.4	0.6	0.4	0.5	0.4	0.3	0.005
Mixed	0.9	1.0	0.9	0.7	0.8	0.9	0.266
Other	1.5	1.4	1.5	1.7	1.3	1.5	0.799
White	91.1	90.4	91.3	90.9	91.6	91.3	0.006

Continuous values given as mean (SD).

BMI, body mass index; bpm, beats per minute; LTL, loge-transformed leucocyte telomere length; WBC, white blood cell count.

**Table 2:** Baseline characteristics according to quintiles of QRS duration

Variable	Full cohort (62 266)	QRS duration quintile levels					
		1st (<86 ms)	2nd (86–90 ms)	3rd (90–94 ms)	4th (94–98 ms)	5th (>98 ms)	P value
Chronological age (years)	56.8 (8.1)	56.7 (8.1)	56.5 (8.1)	56.6 (8.1)	56.6 (8.2)	57.4 (8.2)	<0.001
Men (%)	46.7	34.5	44.4	49.2	54.7	59.1	<0.001
WBC (mmol/L)	7.1 (2.0)	7.1 (2.1)	7.1 (2.0)	7.0 (1.8)	7.0 (1.8)	7.0 (2.3)	<0.001
Height (cm)	168.9 (9.2)	166.2 (8.8)	168.4 (9.1)	169.4 (9.1)	170.6 (9.2)	171.6 (9.1)	<0.001
BMI	27.4 (4.7)	27.1 (4.6)	27.5 (4.7)	27.5 (4.7)	27.4 (4.7)	27.5 (4.9)	<0.001
LTL	0.0 (1.0)	0.0 (1.0)	0.0 (1.0)	0.0 (1.0)	0.0 (1.0)	0.0 (1.0)	<0.001
Heart rate (bpm)	71.2 (12.0)	73.4 (12.2)	71.9 (11.8)	71.0 (11.7)	70.0 (11.8)	68.3 (11.8)	<0.001
Coronary artery disease (%)	3.4	2.2	2.9	3.1	3.6	5.7	<0.001
Hypertension (%)	53.1	50.0	52.9	54.2	53.6	56.4	<0.001
Active smoker (%)	9.4	8.9	9.3	9.8	9.9	9.5	0.051
Diabetes mellitus (%)	5.5	5.0	5.3	5.5	5.5	6.2	<0.001
Ancestry (%)							
Asian	3.1	4.0	3.4	2.9	2.4	2.0	<0.001
Black	2.9	3.8	3.4	2.9	2.3	1.4	<0.001
Chinese	0.4	0.6	0.6	0.4	0.4	0.2	<0.001
Mixed	0.8	1.0	1.0	0.8	0.7	0.6	<0.001
Other	1.5	1.8	1.6	1.5	1.3	0.9	<0.001
White	91.3	88.9	90.0	91.6	92.8	94.9	<0.001

Continuous values given as mean (SD).

BMI, body mass index; bpm, beats per minute; LTL, loge-transformed leucocyte telomere length; WBC, white blood cell count.

**Table 3:** Baseline characteristics for the pacemaker implantation cohort

	Full cohort (420 071)	Incident pacemaker implantation		P value
		Yes (7169)	No (412 902)	
Chronological age (years)	56.5 (8.0)	56.4 (8.0)	62.0 (6.0)	<0.001
Men (%)	46.0	45.6	68.4	<0.001
WBC (mmol/L)	6.9 (2.0)	6.9 (2.0)	7.1 (1.9)	<0.001
LTL	0.0 (1.0)	0.0 (1.0)	−0.2 (1.0)	<0.001
Coronary artery disease (%)	3.8	3.7	13.7	<0.001
Hypertension (%)	53.2	52.8	75.2	<0.001
Active smoker (%)	10.6	10.6	9.8	0.032
Diabetes mellitus (%)	5.3	5.2	12.2	<0.001
Ancestry (%)				
Asian	2.0	2.0	2.2	0.145
Black	1.5	1.6	0.6	<0.001
Chinese	0.3	0.3	0.2	0.014
Mixed	0.6	0.6	0.3	0.003
Other	0.9	0.9	0.7	0.067
White	94.7	94.6	96.0	<0.001

Continuous values given as mean (SD).

LTL, loge-transformed leucocyte telomere length; WBC, white blood cell count.

### Observational associations between LTL, ECG conduction measurements and incident pacemaker implantation

In the minimally adjusted model (sex, height, BMI and heart rate), LTL was significantly associated with PR interval (beta=0.18 ms per 1 SD decrease in log_e_ LTL, 95% CI: 0.02 to 0.34 ms, p=0.029), but not QRS duration (table 4). The association between LTL and PR interval was not attenuated and remained statistically significant after additional adjusting for cardiovascular risk factors and coronary artery disease (table 4). In the sensitivity analyses, no important changes were observed in the magnitude and direction of the effect when additionally adjusting for vagal tone or excluding individuals exposed to beta and calcium blockers (online supplemental tables 4 and 5). In the pacemaker cohort, shorter LTL was associated with a higher risk of future pacemaker implantation when adjusting for age and sex (HR per 1 SD decrease in log_e_ LTL: 1.04; 95% CI: 1.02 to 1.07; p<0.001). The association remained significant with little attenuation after additional adjusting for cardiovascular risk factors (type 2 diabetes mellitus, current smoking and hypertension) and coronary artery disease (table 4). No evidence was found that the association was affected by beta and calcium blockers in the sensitivity analysis (online supplemental table 5). To put the effect sizes for LTL in perspective, we also calculated the effect size of (chronological) age on PR interval: After adjusting for the same cardiovascular risk factors, 1 year increase in age was found to have a similar effect size for PR interval compared with 1 SD reduction in LTL (0.19 ms per 1 year increase in age, 95% CI: 0.17 to 0.21, p<0.001). For pacemaker implantation, the HR was 1.10 per 1-year increase in age (95% CI: 1.10 to 1.11, p<0.001). The HR of 1 SD decrease in LTL (HR 1.03) was therefore comparable with ~3.8 months older age.

**Table 4:** Multivariable regression results for the association between leucocyte telomere length and PR interval, QRS duration and incident pacemaker implantation

PR interval			
Model	Beta	95% CI	P value
Adjusted for sex, age, height, BMI and heart rate	0.18	0.02 to 0.34	0.029
… + T2DM, current smoking, hypertension and CAD	0.19	0.03 to 0.35	0.021

QRS duration			
Model	Beta	95% CI	P value
Adjusted for sex, age, height, BMI and heart rate	0.04	−0.02 to 0.11	0.152

Incident pacemaker implantation			
Model	HR	95% CI	P value
Adjusted for sex and age	1.04	1.02 to 1.07	<0.001
… + T2DM, current smoking, hypertension and CAD	1.03	1.01 to 1.06	0.012

Effect sizes and HRs are expressed per 1 SD decrease in leucocyte telomere length.

BMI, body mass index; CAD, prevalent coronary artery disease; T2DM, type 2 diabetes mellitus.

### MR analyses

Using 130 genetic variants independently associated with LTL as instruments^8^ in the MR analysis, we found nominal significant associations for a direct effect from LTL to PR interval and pacemaker implantation with consistent directions of effect (0.74 ms increase in PR interval per 1 SD shorter LTL, 95% CI: 0.01 to 1.47; and HR of 1.16 for pacemaker implantation per 1 SD shorter LTL, 95% CI: 1.00 to 1.34, table 5). This HR was comparable with 18.7 months older age (as shown above, HR of age is 1.10 per 1-year increase). No evidence of horizontal pleiotropy was found in the MR-Egger analyses and while effect sizes remain consistent across the sensitivity analyses the nominal level of significance was lost when applying the weighted median and contamination mixture methods in the sensitivity analysis (table 5).

**Table 5:** Mendelian randomisation associations between leucocyte telomere length (LTL) and PR interval and pacemaker implantation

MR method	PR interval		Pacemaker implantation	
	Beta (95% CI)	P	HR (95% CI)	P value
IVW	0.74 (0.01 to 1.47)	0.048	1.16 (1.00 to 1.34)	0.046
Weighted median	0.73 (−0.19 to 1.65)	0.122	1.19 (0.97 to 1.48)	0.100
ConMix	0.46 (−0.16 to 1.05)	0.144	1.21 (0.97 to 1.42)	0.088
Egger intercept		0.936		0.599

Associations per 1 SD shorter LTL.

ConMix, contamination mixture; IVW, inverse variance weighted; MR, mendelian randomisation.

## Discussion

This work represents the largest population-based observational study to date to examine the impact of cellular ageing, measured by LTL, on the deterioration of cardiac conduction in humans. Our main findings are that in a middle-aged population: (1) LTL is associated with PR interval, but not QRS duration, (2) LTL is associated with incident pacemaker implantation and (3) evidence from MR analysis suggest the observed associations might be causal, where shorter LTL increases PR interval and the risk of pacemaker implantation.

### Association of LTL with ECG measures of cardiac conduction

Our work confirms previous data of prolongation of PR interval and QRS duration with increasing ageing.^2 21^ However, only a very small number of studies have investigated the implications of biological ageing. Most recently, von Falkenhausen *et al*^22^ found no effect of LTL on PR interval and QRS duration in 2575 participants from the community-based KORA (Kooperative Gesundheitsforschung in der Region Augsburg) study after accounting for age, sex, height and BMI. With ~60 000 participants, our study was considerably larger and better powered to detect any potential association. It is likely that this has enabled us to detect the association between LTL and PR interval, as the adjusted effect size was very small (eg, <1 ms increase in PR interval per 1 SD LTL shortening). In addition, von Falkenhausen *et al* did not adjust for heart rate, which might have potentially diluted their results as PR interval is known to vary with heart rate.^23^ Our direction of effect was consistent with that reported in the KORA study. Like von Falkenhausen, we did not find evidence that LTL was associated with QRS duration. There may be different explanations compatible with these findings. For example, age-related processes might be different for AV and intraventricular conduction. These processes may include diminished dromotropic effect of catecholamines on AV junctional tissues. For example, ageing has been associated with diminished chronotropic and inotropic responses to catecholamines.^24 25^ However, in the sensitivity analysis, we found no evidence to suggest that vagal tone could explain the association between LTL and PR interval. Alternative explanations include structural changes that occur within the AV junction with advanced age,^26^ whereas QRS duration may depend more on age-related functional and structural changes of the ventricular myocardium, for example, due to fibrosis.^27 28^ Interestingly, we have recently demonstrated a (causal) association between LTL and cardiac dimensions and function using cardiac imaging,^29^ it might be that these processes have little impact on ventricular conduction and QRS duration. It is also worth noting that the PR interval represents the total transmission time through both atria, the AV node, His bundle, and bundle branches to the onset of ventricular activation via the Purkinje system, and an increased conduction delay can occur at any of these sites.^30^ Alternatively, the lack of association may also simply reflect that fact that QRS duration shows less variation with age compared with PR interval, making it harder to measure the association with LTL.

### Association of LTL with incident pacemaker implantation

We further enhance our understanding of the role of cellular ageing in the deterioration of the cardiac conduction system by demonstrating that shorter LTL is associated with higher risk of incident pacemaker implantation. This provides an additional line of evidence that shows that cellular ageing may not only be associated with AV conduction (PR interval) as a continuous variable, but also the actual clinically significant pathophysiology that underlies the deterioration of the cardiac conduction system, which requires pacemaker intervention. The exact mechanisms remain unclear, but our results suggest that they may include processes linked to accelerated ageing.

### Causal relationship between LTL and cardiac conduction

In this work, we also explored, for the first time, the causal relationship between LTL and cardiac conduction disease. Results from the MR analysis hint at a potential trend indicating that a reduction in LTL may cause an increase in the PR interval and a higher risk of incident pacemaker implantation. However, the observed associations did not retain statistical significance in sensitivity analyses, likely due to the limited study power (<10% power to detect any association). This contrast with our findings for other age-related cardiovascular diseases such as coronary artery disease and heart failure.^8 29^ Whether the LTL associations are causal or not, the (causal) mechanisms for the biological age-associated increase in PR interval and a higher risk of pacemaker implantation remain therefore to be further investigated.

### Clinical implications

Although associations between LTL and cardiac conduction were statistically significant, it is important to interpret this in the context of the study’s high power to detect even modest associations. The observed effect sizes in this work were small and may therefore not necessarily imply clinical importance. For example, the observed 3% increased risk of pacemaker implantation for 1 SD shortening in LTL was estimated to be equivalent to a 3.8 month increase in (chronological) age within the observational analysis (see Results section), and yet in our MR this estimate increases to an estimated 18.7 months older age. However, the observational associations between LTL and PR interval and pacemaker implantation were independent of other cardiovascular risk factors potentially suggesting that telomere biology, and perhaps cellular ageing in general, may contribute uniquely to the pathophysiology of the cardiac conduction system. This may offer potential for improved risk stratification and novel insights into disease mechanisms beyond (chronological) age and other traditional cardiovascular risk factors. Results also support the growing body of evidence linking telomere biology to cardiovascular health outcomes.^8 29^ Further research is needed to explore these associations in more detail, particularly to understand the underlying mechanisms and to investigate other markers of ageing in relation to cardiac conduction system pathophysiology.

### Strengths and limitations

Our study has several strengths, including access to the largest sample size to date with long-term follow-up to study the association between LTL, PR interval, QRS duration and pacemaker implantation. Nevertheless, several limitations need to be acknowledged. First, analogous to previous studies, this is a post hoc analysis on a database where conduction disease was not the primary outcome of interest. We applied a rather conservative case definition for pacemaker implantation, which may have caused an underestimation of the actual rate and therefore attenuated the effect estimations. Second, there is a ‘healthy volunteer’ selection bias in the UK Biobank with the participants being older and healthier than the UK general population. Third, the majority of our cohort (97%) is of white ancestry, which may limit the generalisability of our findings in under-represented ethnicities. Fourth, we cannot fully rule out that the observed associations might be affected by residual confounding from undetected cardiovascular disease or unmeasured medication use. However, given that our cohort was rather healthy and that there was no supportive evidence for confounding by beta and calcium blocker medication use, we believe the impact is likely to be limited. Finally, TL was quantified in blood leucocytes, which may not reflect the TL of conduction tissue or other cellsTL.

In conclusion, in this long-term prospective cohort study, we studied the association of LTL with two related but distinct aspects of the cardiac conduction system. One aspect reflected measures of electrical conduction, where we demonstrate that shorter LTL is associated with increased AV conduction delay. The other aspect reflected on incident pacemaker implantation for a bradycardia indication, a significant clinical outcome of cardiac conduction disorder, where we demonstrate that shorter LTL is associated with a higher risk of pacemaker implantation. Combined, results suggest a potential role of cellular ageing as a mechanistic pathway for age-related conduction disease, providing insights into novel risk stratification approaches and therapeutic targets for conduction disease. In this context, future work may focus on alternative measures of biological ageing.

## Supplementary material

10.1136/heartjnl-2024-324875 online supplemental file 1

10.1136/heartjnl-2024-324875 online supplemental file 2

## Data Availability

Data may be obtained from a third party and are not publicly available.
