# Supplementary material for: Leucocyte telomere length and conduction system ageing
Source: Heart. 2024 Dec 17;111(7):e324875. doi: 10.1136/heartjnl-2024-324875 (PMC12015050; doi:10.1136/heartjnl-2024-324875)

Supplemental Figure 1. Assessment of proportional hazard assumptions: leukocyte telomere length (LTL) – incident pacemaker implantation


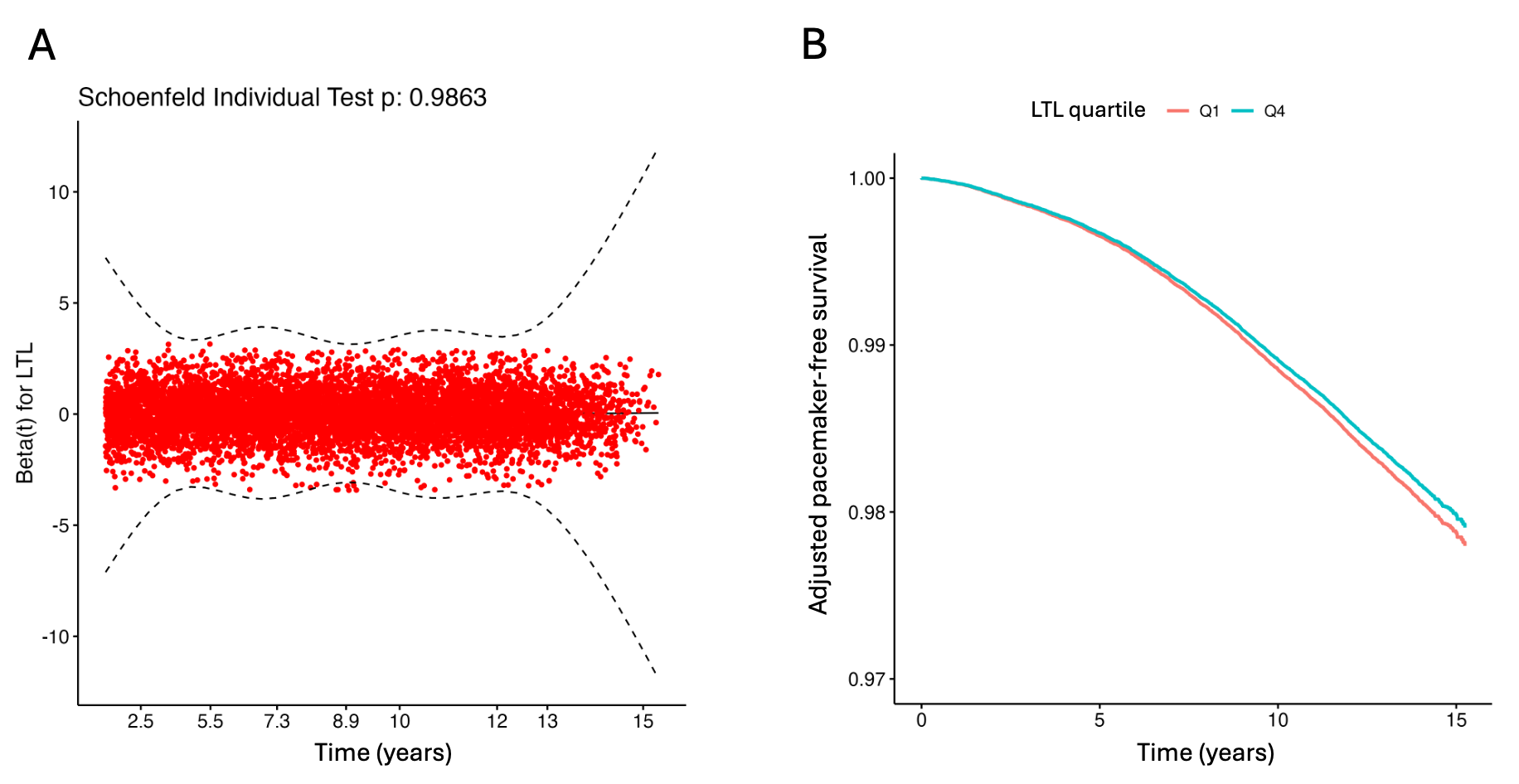

Supplement: online supplemental file 1 [file heartjnl-111-7-s001.docx]
