# Supplementary material for: Leucocyte telomere length and conduction system ageing
Source: Heart. 2024 Dec 17;111(7):e324875. doi: 10.1136/heartjnl-2024-324875 (PMC12015050; doi:10.1136/heartjnl-2024-324875)
Supplement: online supplemental file 2 [file heartjnl-111-7-s002.docx]

| Supplemental Table 1: Codes used to identify individuals with pre-existing pacemaker. | | |
| --- | --- | --- |
|  |  |  |
| source | code | description |
| OPSC-4 | K60.1 | Implantation of intravenous cardiac pacemaker system NEC |
| OPSC-4 | K60.2 | Resiting of lead of intravenous cardiac pacemaker system |
| OPSC-4 | K60.3 | Renewal of intravenous cardiac pacemaker system |
| OPSC-4 | K60.4 | Removal of intravenous cardiac pacemaker system |
| OPSC-4 | K60.5 | Implantation of intravenous single chamber cardiac pacemaker system |
| OPSC-4 | K60.6 | Implantation of intravenous dual chamber cardiac pacemaker system |
| OPSC-4 | K60.8 | Other specified cardiac pacemaker system introduced through vein |
| OPSC-4 | K60.9 | Unspecified cardiac pacemaker system introduced through vein |
| OPSC-4 | K61.1 | Implantation of cardiac pacemaker system NEC |
| OPSC-4 | K61.2 | Resiting of lead of cardiac pacemaker system NEC |
| OPSC-4 | K61.3 | Renewal of cardiac pacemaker system NEC |
| OPSC-4 | K61.4 | Removal of cardiac pacemaker system NEC |
| OPSC-4 | K61.5 | Implantation of single chamber cardiac pacemaker system |
| OPSC-4 | K61.6 | Implantation of dual chamber cardiac pacemaker system |
| OPSC-4 | K61.8 | Other specified cardiac pacemaker system |
| OPSC-4 | K61.9 | Unspecified cardiac pacemaker system |
| ICD10 | Z45.0 | Adjustment and management of cardiac pacemaker |
| ICD10 | Z95.0 | Presence of cardiac pacemaker |
| ICD9 | V450 | Cardiac pacemaker in situ |
|  |  |  |
| *OPSC-4: Office of Population Censuses and Surveys Classification of Interventions and Procedures version 4; ICD: International Classification of Diseases* | | |

| Supplemental Table 2: Codes used to identify individuals with de-novo pacemaker implantation. | | |
| --- | --- | --- |
|  |  |  |
| source | code | description |
| OPSC-4 | K60.1 | Implantation of intravenous cardiac pacemaker system NEC |
| OPSC-4 | K60.5 | Implantation of intravenous single chamber cardiac pacemaker system |
| OPSC-4 | K60.6 | Implantation of intravenous dual chamber cardiac pacemaker system |
| OPSC-4 | K60.8 | Other specified cardiac pacemaker system introduced through vein |
| OPSC-4 | K60.9 | Unspecified cardiac pacemaker system introduced through vein |
| OPSC-4 | K61.1 | Implantation of cardiac pacemaker system NEC |
| OPSC-4 | K61.5 | Implantation of single chamber cardiac pacemaker system |
| OPSC-4 | K61.6 | Implantation of dual chamber cardiac pacemaker system |
| OPSC-4 | K61.8 | Other specified cardiac pacemaker system |
| OPSC-4 | K61.9 | Unspecified cardiac pacemaker system |
|  |  |  |
| Individuals who had their pacemaker excluded during follow-up where excluded from the analysis unless they had a re-implantation. *OPSC-4: Office of Population Censuses and Surveys Classification of Interventions and Procedures version 4* | | |

| Supplemental Table 3: Definition of coronary artery disease | | |
| --- | --- | --- |
|  |  |  |
| Code type | Code | Definitions |
| ICD10 | I21 | Acute myocardial infarction |
| ICD10 | I22 | Subsequent myocardial infarction |
| ICD10 | I23 | Certain current complications following acute myocardial infarction. |
| ICD10 | I24 | Other acute ischaemic heart diseases. |
| ICD10 | I25 | Chronic Ischemic Heart Disease |
| ICD9 | 410 | Acute myocardial infarction |
| ICD9 | 411 | Other acute and subacute forms of ischemic heart disease |
| ICD9 | 412 | Old myocardial infarction |
| ICD9 | 414 | Other forms of chronic ischemic heart disease |
| OPCS4 | K40 | Saphenous vein graft replacement of coronary artery |
| OPCS4 | K41 | Other autograft replacement of coronary artery |
| OPCS4 | K42 | Allograft replacement of coronary artery |
| OPCS4 | K43 | Prosthetic replacement of coronary artery |
| OPCS4 | K44 | Other replacement of coronary artery |
| OPCS4 | K45 | Connection of thoracic artery to coronary artery |
| OPCS4 | K46 | Other bypass of coronary artery12 |
| OPCS4 | K49 | Transluminal balloon angioplasty of coronary artery |
| OPCS4 | K501 | Percutaneous transluminal laser coronary angioplasty |
| OPCS4 | K75 | Percutaneous transluminal balloon angioplasty and insertion of stent into coronary artery |
| Self-reported medical conditions | 1 | Heart Attack |
| Self-reported operations | 1070 | coronary angioplasty (ptca) +/- stent |
| Self-reported operations | 1095 | coronary artery bypass grafts (cabg) |
| Self-reported operations | 1523 | triple heart bypass |
|  |  |  |
| *OPSC-4: Office of Population Censuses and Surveys Classification of Interventions and Procedures version 4; ICD: International Classification of Diseases* | | |

Supplemental Table 4: Multivariable regression results for the association between leukocyte telomere length and PR interval after additionally adjusting for vagal tone (hear rate recovery) in post-hoc sensitivity analysis

|  |  |  |
| --- | --- | --- |
| Model | beta | 95% CI |
| *Adjusted for sex, age, height, BMI, resting heart rate, T2DM, current smoking, hypertension, and CAD* | 0.13 | -0.06 - 0.31 |
| *…+ heart rate recovery* | 0.12 | -0.06 - 0.30 |

*N=47,755 individuals with heart rate recovery measured.*

Supplemental Table 5: Multivariable regression results for the association between leukocyte telomere length, PR interval, and pacemaker implantation after excluding for beta and calcium antagonists in post-hoc sensitivity analyses.

| ***PR interval*** | Original analysis, N=59,868 | | After excluding for beta-blocker & calcium antagonists, N=55,829 | |
| --- | --- | --- | --- | --- |
| Model | Beta | 95% CI | beta | 95% CI |
| *Adjusted for sex, age, height, BMI, resting heart rate, T2DM, current smoking, hypertension, and CAD* | 0.19 | 0.03 - 0.36 | 0.17 | 0.01 - 0.34 |

| ***Incident pacemaker implantation*** | Original analysis, N = 420,071 | | After excluding beta-blocker and calcium antagonists,  N = 385,811 | |
| --- | --- | --- | --- | --- |
| Model | Hazard ratio | 95% CI | Hazard ratio | 95% CI |
| *Adjusted for sex, age, T2DM, current smoking, hypertension, and CAD* | 1.03 | 1.01 - 1.06 | 1.04 | 1.01 - 1.07 |
